# 

**Published:** 1918-11-30

**Authors:** 


### THE COLLEGE OF NURSING

**(Limited by Guarantee).**

EDINBURGH CENTRE.

### Miss Newbigin on "The Renewal of Life."

THE lecture-room in the City Chambers, Edinburgh, was crowded on Tuesday, November 19, when the second lecture of the series arranged under the auspices of the College of Nursing by the National Council for Combating Venereal Diseases was held. Miss Sandford introduced the speaker, Miss Marion I. Newbigin, D.Sc. Though a first-class biologist, Dr. Newbigin is probably best known as the editor of the *Scottish Geographical Magazine*, and the author of various geographical works. There was something reminiscent of Maeterlinck's "Intelligence des Fleures" in the opening of the lecture, for it was from the vegetable kingdom, and indeed from the homely vegetable marrow that Dr. Newbigin drew her first illustrations of sexual union and reproduction. From the grains of wheat whose pollen is blown by the wind "whither it listeth," it is a far cry to the human embryo, but with the aid of diagrams and photographs thrown on the screen the whole field was traversed. The audience saw how bees and humming-birds fertilise the flowers as they fly from plant to plant. They were shown how the volvox and the green-fly reproduce themselves in the happy days of summer, and how only by the more laborious and costly method of sexual reproduction can they bring forth offspring strong enough to resist the stern conditions of winter. Dr. Newbigin analysed the typical male and female cells—the first thin and hungry, propelling itself rapidly through water by whiplike movements, the second larger, rounder, and more stationary; and demonstrated by means of diagrams the method of division of the chromatin thread—that mysterious substance by which alone a father can transmit his nature to his children. They were shown the wasteful method of the breeding of frogs, and saw how, by the provision of bags of food in their eggs, even certain mother fish showed more care for their young. The life history of the chick was given in some detail, and the audience heard incidentally how on the Forth the firing of big guns had crippled a large number of chickens before they were hatched, despite the protection of the allantois. Lastly, we looked upon the human embryo.

In conclusion, Dr. Newbigin touched on some of the wider problems of heredity. She dwelt upon the necessity of "the infusion of new blood," and pointed out how instinctively all peoples, whether civilised or savage, abhor the idea of sexual union between persons very closely related. Intermarriage, of course, did take place throughout the generations, but in excess it was pernicious. The Kaiser, whose boast used to be that he knew more of his ancestors than any man living, could only count 275 of the twelfth generation out of a possible 4,096, who should theoretically all have been living about the beginning of the sixteenth century.

Miss Cumming, R.R.C., the new matron of the Longmore Hospital, thanked Dr. Newbigin on behalf of the audience for her most interesting lecture.

### Matrons' and Sisters' Appointments.

BOWLING PARK HOSPITAL, BRADFORD.—Miss Fanny M. Collin has been appointed surgical-ward sister. She was trained at the North Evington Infirmary, Leicester, where she has since been staff nurse, medical-ward sister, theatre, and surgical-ward sister. She has also been ward sister at Fulham Infirmary, and night sister at Fulham Military Hospital.

CHELSEA HOSPITAL FOR WOMEN, S.W.—Miss Alice Thorne has been appointed assistant matron. She was trained at the London Homœopathic Hospital, has been sister at the Bromley Cottage Hospital, theatre and ward sister at the Women's Hospital, Soho Square, casualty, gynæcological, and military ward sister at Charing Cross Hospital, and has served at Salonika with the Scottish Women's Hospitals.

COTTAGE HOSPITAL, BALLYMENA, CO. ANTRIM.—Miss Ethel McMath has been appointed matron. She was trained at the Royal Victoria Hospital, Belfast, and has been sister at the Fitzroy Private Hospital, Belfast, and at the Ulster, Volunteer Hospital, Belfast.

EDITH CAVELL HOME OF REST FOR NURSES, HASLEMERE.—Miss Julia Hurlston has been appointed matron. She was trained at St. Bartholomew's Hospital, has been sister at the Victoria Hospital for Children, Chelsea, at St. Peter's Hospital, and sister-in-charge of Minifield House, Gullane. She has also done private nursing.

GENERAL HOSPITAL, NOTTINGHAM.—Miss G. B. Mills has been appointed children's ward sister. She was trained at the District Infirmary and Children's Hospital, Ashton-under-Lyne; has been sister of the medical wards at the Victoria Hospital, Hull; sister of the men's medical ward, Warneford Hospital, Leamington Spa; and night sister at the Royal National Orthopædic Hospital, London.

HOSPITAL FOR EPILEPSY AND PARALYSIS, MAIDA VALE.—Miss Ethel Wakeman and Miss Olive Despard have been appointed theatre and ward sister respectively. Miss Wakeman was trained at the Herefordshire General Hospital, later being night sister there, and ward sister of the Women's Surgical Ward, Princess Alice Memorial Hospital, Eastbourne. Miss Despard was trained at the David Lewis Northern Hospital, Liverpool, and at the Abingdon Joint Hospital, Berks. She has done private nursing, and has served in the Queen Alexandra Imperial Military Nursing Service Reserve.

INFANTS' HOSPITAL, VINCENT SQUARE, WESTMINSTER.—Miss Ethel M. Franklin has been appointed night sister. She was trained at the London Homœopathic Hospital, and has been theatre sister at the County Hospital, Bedford.

INFECTIOUS DISEASES HOSPITAL, NEAR GLASGOW.—Miss Jenny C. G. Macfarlane has been appointed matron. She was trained at St. George's Hospital, London, and at Ruchill Fever Hospital, Glasgow. She has been sister at the former institution, at the Royal Infirmary, Perth, and assistant matron of Knightswood Hospital, Glasgow.

INFECTIOUS DISEASES HOSPITAL, PORTSMOUTH. — Miss Gabrielle Cravola has been appointed sister. She was trained at Croydon Infirmary, has been nurse at the Park Fever Hospital, staff nurse at Chelsea Hospital for Women, and has done private nursing in Canada.

ISOLATION HOSPITAL, BASINGSTOKE.—Miss E. C. Burdfield has been appointed nurse-matron. She was trained at Croydon Borough Hospital, and has been assistant matron at the Sanatorium, Southall.

ISOLATION HOSPITAL, WIMBLEDON.—Miss Fanny Fairbrother has been appointed sister. She was trained at the Hampstead General Hospital, has been ward sister at the Borough Sanatorium, Sunderland, and sister at the City Hospital, Lodge Moor, Sheffield. She has also done private and district nursing.

LAKE HOSPITAL, ASHTON-UNDER-LYNE.—Mrs. Nellie McKann, Mrs. Nellie Frost, and Miss Rachel Bark have been appointed sisters. They were all trained at the above-named hospital, where they have been staff nurses.

MORNINGFIELD HOSPITAL, ABERDEEN.—Miss Margaret Steele Fraser has been appointed sister. She was trained at the West Fife Hospital, Dunfermline, and has been staff nurse at the Lord Derby War Hospital, Warrington.

NATIONAL HOSPITAL FOR THE PARALYSED AND EPILEPTIC, QUEEN SQUARE.—Miss J. Parker has been appointed assistant matron. She was trained at the County Infirmary, Londonderry, and has been ward sister at the above hospital. Miss Margaret Buckle has been appointed ward sister. She was trained at Leeds General Infirmary, and has since nursed at the King George Hospital, S.E.

QUEEN ALEXANDRA'S MILITARY NURSING SERVICE FOR INDIA. The Misses E. E. Bott, Vera Francis, G. Harvey James, F. G. Warren, E. M. McPherson, and Mrs. A. L. Blomfield Dickson have been appointed to this Service.

ROYAL HANTS COUNTY HOSPITAL, WINCHESTER.—Miss Dorcas A. Willey has been appointed sister. She was trained at Leicester Royal Infirmary, and has since been sister at the Royal Victoria Hospital, Dover; theatre sister at 19th General Hospital, Alexandria, at the 2nd Birmingham War Hospital, and at the Dreadnought Seamen's Hospital, Greenwich.

ROYAL SOUTH HANTS HOSPITAL, SOUTHAMPTON.—Miss E. Bowker has been appointed ward sister. She was trained at the Royal Infirmary, Huddersfield, has been staff nurse at the Manor War Hospital, Epsom, and also under the Queen Alexandra Imperial Military Nursing Service Reserve. Miss Bowker holds the certificate of the Central Midwives Board.

ROYAL WATERLOO HOSPITAL, S.E.—Miss Ida M. Coleclough has been appointed sister. She was trained at the Leicester Royal Infirmary, and has been night sister at the Victoria Hospital for Children, Chelsea.

SAMARITAN FREE HOSPITAL FOR WOMEN, MARYLEBONE ROAD.—Miss McIntosh has been appointed home sister. She was trained at the Royal Hospital, Sheffield, and at Queen Charlotte's Lying-in Hospital, and has held the posts of ward and night sister.

SOMERSET COUNTY NURSING ASSOCIATION.—Miss Florence Gane has been appointed Inspector of Midwives and Superintendent of this Association affiliated to the Queen's Institute. She was trained at the Norfolk and Norwich Hospital, and took her midwifery training at the London Hospital. She holds the certificates of the Central Midwives Board and the Royal Sanitary Institute. She has held posts under the Sheffield Borough Council, the London County Council, and has been Inspector of Midwives for the Lancashire County Council.

TARDEBIGG HOSPITAL, REDDITCH.—Miss Francis E. Driver has been appointed night sister. She was trained at the Manchester Royal Infirmary, and has held posts at the V.A.D. Hospital, Topsham, South Devon; Elm Bank Hospital, Eccles; Red Cross Hospital, Darley Dale, Derbyshire; and at the Linden Lea Auxiliary Military Hospital, Brooklands.

UNION INFIRMARY, BROMSGROVE.—Miss Leah Lillie Simmonds has been appointed superintendent nurse. She was trained at the Dudley Road Infirmary, Birmingham, and has been superintendent nurse at the Union Infirmary, Northampton, and under the Oswestry Incorporation.

WAKEFIELD MATERNITY HOSPITAL.—Miss Charlotte Dickson has been appointed matron. She was trained at the West London Hospital, Hammersmith, and at Queen Charlotte's Hospital. She has held posts at the Queen's Hospital for Children, Bethnal Green, Hampstead General Hospital, and the Samaritan Free Hospital, and is at present home sister at the Great Ormond Street Children's Hospital.

WELSH NATIONAL HOSPITAL, NETLEY.—Miss Helen Mary Akerigg has been appointed matron. She was trained at Addenbrooke's Hospital, Cambridge, where latterly she has been assistant matron. She has also been night superintendent and sister at St. George's Hospital, London; acting matron of the Cray Valley Hospital; matron of Fleet Cottage Hospital and of Clayton Court Auxiliary Hospital, Liss; and sister under the Territorial Force Nursing Service at the 1st Southern Hospital, Birmingham.

**NOTE.—Particulars of Appointments for insertion in this column will be welcomed.**

